# Promoting parent-child relationships and preventing violence via home-visiting: a pre-post cluster randomised trial among Rwandan families linked to social protection programmes

**DOI:** 10.1186/s12889-020-08693-7

**Published:** 2020-05-06

**Authors:** Theresa S. Betancourt, Sarah K. G. Jensen, Dale A. Barnhart, Robert T. Brennan, Shauna M. Murray, Aisha K. Yousafzai, Jordan Farrar, Kalisa Godfroid, Stephanie M. Bazubagira, Laura B. Rawlings, Briana Wilson, Vincent Sezibera, Alex Kamurase

**Affiliations:** 1 Boston College School of Social Work, McGuinn Hall 106M, 140 Commonwealth Avenue, Chestnut Hill, MA 02467 USA; 2 Department of Epidemiology, Harvard School of Public Health, Boston, MA USA; 3 Women’s Study Research Center, Brandeis University, Waltham, MA USA; 4 Department of Global Health and Population, Harvard School of Public Health, Boston, MA USA; 5 FXB Rwanda, Kigali, Rwanda; 6 The World Bank, Washington, DC USA; 7 College of Medicine and Health Sciences, University of Rwanda, Kigali, Rwanda

**Keywords:** Home-visiting, Early childhood development (ECD), Violence, Social protection, Father engagement, Poverty

## Abstract

**Background:**

*Sugira Muryango* is a father-engaged early child development and violence-prevention home-visiting programme delivered by trained lay workers. This cluster-randomised trial evaluates whether families living in extreme poverty (*Ubudehe* 1, the poorest category in the Government of Rwanda’s wealth ranking) who receive *Sugira Muryango* in combination with a government-provided social protection programme demonstrate greater responsive, positive caregiving, nutrition, care seeking, hygiene, and father involvement compared with control families receiving usual care (UC).

**Methods:**

Using detailed maps, we grouped closely spaced villages into 284 geographic clusters stratified by the type of social protection programmes operating in the village clusters; 198 clusters met all enrolment criteria. *Sugira Muryango* was delivered to *n* = 541 families in 100 treatment clusters with children aged 6–36 months living in extreme poverty. We assessed changes in outcomes in intervention and *n* = 508 UC control families using structured surveys and observation. Analyses were intent to treat using mixed models to accommodate clustering.

**Results:**

Families receiving *Sugira Muryango* improved on core outcomes of parent-child relationships assessed using the Home Observation for Measurement of the Environment (Cohen’s *d* = 0.87, 95% CI: 0.74, 0.99) and the Observation of Mother-Child Interaction (Cohen’s *d* = 0.29, 95% CI: 0.17, 0.41). We also saw reductions in harsh discipline on items from the UNICEF MICS (OR = 0.30: 95% CI: 0.19, 0.47) and in violent victimisation of female caregivers by their partners (OR = 0.49, 95% CI: 0.24, 1.00) compared with UC. Moreover, children in families receiving SM had a 0.45 higher increase in food groups consumed in the past 24 h (Cohen’s *d* = 0.35, 95% CI: 0.22, 0.47), increased care seeking for diarrhoea (OR = 4.43, 95% CI: 1.95, 10.10) and fever (OR = 3.28, 95% CI: 1.82, 5.89), and improved hygiene behaviours such as proper treatment of water (OR = 3.39, 95% CI: 2.16, 5.30) compared with UC. Finally, *Sugira Muryango* was associated with decreased caregiver depression and anxiety (OR = 0.58, 95% CI: 0.38, 0.88).

**Conclusions:**

*Sugira Muryango* led to improvements in caregiver behaviours linked to child development and health as well as reductions in violence.

**Trial registration:**

ClinicalTrials.gov number NCT02510313.

## Background

Children living in poverty face multiple risks to healthy development including malnutrition, illness, under-stimulating environments, and harsh discipline [1]. Addressing these adversities is critical during early childhood when ongoing neural development makes the brain particularly plastic to environmental influences and the rapid achievement of developmental milestones gives rise to cognitive and emotional changes occurring at a speed unparalleled in any other developmental period [2]. Social protection programmes target poor households where early child development (ECD) deficits are concentrated, such as in poor rural settings, where income support can increase investment in dietary diversity, hygiene, and responsive caregiving [3]. Previous ECD interventions conducted in low- and middle-income countries (LMICs) demonstrate the value of parenting interventions in improving children’s health and development [4, 5]. Moreover, integrated interventions that build parenting content into other interventions such as social protection and nutrition programmes may offer opportunities for synergistic effects on the home environment and parent-child relationships [6]. For example, engagement and education of caregivers can further improve child health and development through behavioural change [3], and engagement of male caregivers in parent-child interaction and caregiving can help reduce family violence [7].

Rwanda is a low-income country in Sub-Saharan Africa. Although poverty rates in Rwanda have declined in recent years, 38% of Rwandans still live in poverty (defined as yearly consumption per adult equal to or less than RWF 159,375/US$207 in 2016), and 16% live in extreme poverty (defined as yearly consumption per adult of equal to or less than RWF 105,064/US$136 in 2016) [8]. These definitions which equal living on less than US$0.60 or US$0.40 per day, respectively, are well below the World Bank definition of extreme poverty as living on less than US$1.90 per day [9]. The Government of Rwanda is addressing poverty-related disparities in ECD via their Vision 2020 Umurenge Program (VUP), which targets nutrition and ECD among the poorest households, offering direct support (unconditional cash transfers), nutrition-sensitive direct support, and public works programming [10]. Rwanda has a robust ECD policy that has been explicitly linked to its *Economic Development and Poverty Reduction Strategy* [11]. In this manner, the VUP poverty reduction programme provides a platform for targeting the most vulnerable households to promote ECD and prevent violence given high rates of difficulties in both of these areas among families in extreme poverty [12].

**Fig. 1:**
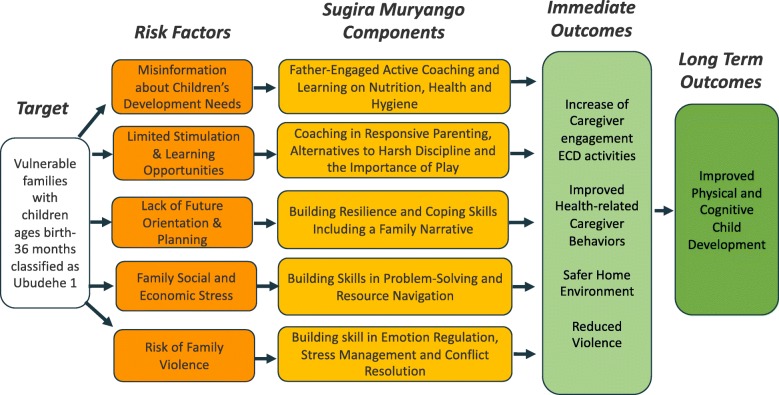
Sugira Muryango Conceptual Model

In this paper, we evaluate the *Sugira Muryango* (Strengthen the Family) home-visiting ECD coaching programme delivered in combination with Rwanda’s VUP support by community-based coaches (CBCs; see Table 1 for selection criteria, training, supervision, and incentives of CBCs). We report data from a cluster randomised trial of 1049 families living in extreme poverty. *Sugira Muryango* comprises five core components (see Theory of Change, Fig. 1): 1) providing psychoeducation on children’s development, nutrition, health, and hygiene promotion; 2) coaching caregivers in active stimulation (play and communication) and responsive parenting to promote “serve-and-return” interactions; 3) reducing family violence via father engagement and improved conflict resolution and parental emotion regulation skills; 4) strengthening problem-solving skills and social support through access to available informal and formal resources; and 5) building skills in positive parenting and coping skills to promote healthy family functioning. These components are tailored towards observed challenges in nurturing care as identified in a report on “Knowledge, Attitudes and Practices Assessment on Early Nurturing of Children” [12]. This report found that family violence including harsh discipline and intimate partner violence were common threats to healthy child development in Rwanda, particularly among poor caregivers who more commonly reported engaging in harsh discipline, including slapping and shouting at the child, compared with families in the higher socioeconomic categories. Moreover, the same report highlighted that fathers in Rwanda are traditionally viewed as the providers for the household rather than active participants in childcare. Key features of emphasis for *Sugira Muryango* are therefore the program’s attention to violence reduction and increasing father engagement in play and caregiving.

**Table 1:** Community Based Coaches (CBCs): training, supervision and incentives

• Selection criteria: a) Live in the beneficiary households they will deliver the intervention to; b) be Rwandan; c) Be aged 18 or older; d) be able to write, read, and count in Kinyarwanda, d) be committed to young children and family values e) have the required amount of time to carry out the *Sugira Muryango* intervention with a select number of households; d) be recommended and approved by local community and authorities	
• Three-week training session delivered by trained supervisors.	
• Training included role-play-based learning, active coaching practice, techniques for engaging fathers, strategies for providing feedback to caregivers on early stimulation, conflict resolution, problem solving, and resource navigation.	
• Supervision provided by *Sugira Muryango* supervisors who had been involved in previous pilot work.	
• Supervision took the form of in-person supervision of the CBCs during the first three weeks of the intervention, and each supervisor shadowed each CBC once in the home. Telephone supervision and peer support groups occurred weekly, and group supervision was held once a month.	
• CBCs audiotaped the home-based sessions, which were reviewed by a supervisor for fidelity monitoring.	

CBCs were stipended according to local practices (28,000 Rwandan Francs per month for a caseload of five families), visiting weekly for a period of three months and participating in all training and supervision

## Methods

### Study design

Between January and September 2018, we conducted baseline and post-intervention assessments of a stratified cluster-randomised trial designed to test *Sugira Muryango’s* effects on promoting ECD and preventing violence among families receiving VUP*.* The trial was conducted within the Nyanza, Ngoma, and Rubavu districts with existing VUP programmes, selected to minimise the overlap with ECD interventions by government or nongovernmental organisations. All families were eligible for one of two versions of the VUP programme: classic public works (cPW), which provides cash for (typically hard) manual labour; or the newer expanded public works (ePW), which provides cash for (typically lighter) labour and also provides access to livestock. All procedures were approved by the Harvard T. H. Chan School of Public Health and Boston College Institutional Review Boards as well as the Rwanda National Ethics Committee, National Committee for Science and Technology, and the National Institute of Statistics of Rwanda.

### Participants

Within selected clusters (described in *Randomisation,* below), families were eligible for inclusion in the study if they 1) belonged to the most extreme level of poverty in the government’s household-ranking system (*Ubudehe* 1) [13] and were eligible for the cPW or ePW programme; 2) had at least one child 6–36 months; and 3) were willing to participate in a home-visiting intervention. The focus of the programme on children aged 6–36 months at enrolment was based on the knowledge that experiences, both social and biological, are particularly important during the first few years of development [3]. Furthermore, *Sugira Muryango* is designed to aid child development to the point where government child care becomes available at age 5; at 12-month follow up in the present study the oldest children will be reaching roughly 4.5 years old. Exclusion criteria were a severe, active crisis in the family such as psychosis or suicide attempts by a caregiver or severe mental impairment in the caregiver, which may have affected the ability to benefit from the programme. All caregivers gave written informed consent for themselves and their eligible children.

The caregiver who stated that he or she knew the child best—typically the mother—provided reports on child development and health, the home environment, caregiver-child relationships, caregiving practices, feeding practices, child health, as well as information about the household, including family composition and assets. All primary male and female caregivers provided self-reports on mental health and victimisation and perpetration of intimate partner violence. Interviews, child assessments, and mother-child observation were conducted in Kinyarwanda in the family’s home. Data were entered on Android tablets by independent local enumerators blinded to intervention status.

### The intervention

*Sugira Muryango* comprises 12 modules (see Table 2) that were delivered by trained, supervised CBCs in the families’ homes, unless contraindicated due to illness or privacy concerns, at a pace of about one module per week (average 90-min sessions) between May and August 2018. ​*Sugira Muryango* ​offers active coaching of caregivers to promote early stimulation, play, nutrition, hygiene, responsive parenting, nonviolent interactions among household members, and engagement of both female and male caregivers. CBCs also help families navigate formal and nonformal resources (e.g. health and nutrition services and social support). *Sugira Muryango* was originally developed and tested in Rwanda for families affected by HIV/AIDS [14]. During previous pilot studies [15], a version focusing on ECD was developed by integrating United Nations International Children’s Emergency Fund (UNICEF) and World Health Organization (WHO) Care for Child Development materials [16]. The CBCs were selected from the local community (see Table 1 for selection criteria, training, supervision, and incentives of CBCs). Primary caregivers participated in the modules in interaction with their child (ren); other caregivers and older children were invited to participate. All visits included a 15-min “active play and communication” session where caregivers received live feedback on parent-child interactions. The usual care (UC) group received VUP services and health services as usual from the Rwandan government and its partners. Intervention and UC families received a stipend (RWF 5000 equivalent to 3 k of rice) after each data collection.

**Table 2:** The twelve *Sugira Muryango* modules

• Module 1: Family Narrative	
• Module 2: The importance of early stimulation and play	
• Module 3: Building early communication skills	
• Module 4: The importance of good nutrition	
• Module 5: The importance of good hygiene	
• Module 6: The importance of good health	
• Module 7: Managing the stresses of parenting and family life	
• Module 8: Resolving conflicts in the home	
• Module 9: The important role that everyone plays in raising a baby well	
• Module 10: Good parenting is better than being born well	
• Module 11: Making the home a place where a baby’s brain can grow	
• Module 12: With a united family, anything is possible	

### Outcomes

Per our theory of change (Fig. 1), immediately following 12 modules of intervention delivered weekly over a 3–4 month period, the primary outcomes were change in parents’ behaviours towards the child including responsive care and play, dietary diversity, care seeking for child health problems, and family violence. Secondary outcomes were caregiver outcomes related to shared decision-making among parents and caregiver mental health, as well as household outcomes related to water, sanitation, and hygiene. Questionnaires were developed and tested during pilot intervention research and followed a forward- and back-translation protocol from English to Kinyarwanda [14].

With regard to child-level outcomes, responsive caregiving was assessed by trained enumerators using three tools, the Observation of Mother-Child Interaction (OMCI) [17], an adapted 43-item version of the infant/toddler Home Observation for Measurement of the Environment (HOME) Inventory, which has previously been used in Uganda [18, 19], and the Multiple Indicator Cluster Survey (MICS) Family Care Indicators (FCI) [20]. The OMCI assesses a five-minute mother-child interaction that is scored according to published guidelines (maximum 57; Cronbach’s α = 0.91). The HOME combines observation of parenting behaviours and household conditions with caregiver report. Items were summed to derive a total score (maximum 43; Cronbach’s α *=* 0.76). The MICS FCI^20^ is a cumulative score of caregivers’ self-reported engagement in stimulating activities such as singing, reading, and counting with the child during the prior 3 days (maximum 6; Cronbach’s α = 0.74). Children’s nutritional intake was assessed by parent-reported dietary diversity reflecting the number of seven food groups (grains, roots, and tubers; legumes and nuts; dairy products; meat, fish, poultry, and organ meats; eggs; vitamin A rich fruits and vegetables; other fruits and vegetables) the child had consumed in the past 24 h [21]. Children’s health status was measured using standard Demographic and Health Surveys (DHS) questions reporting the prevalence of diarrhoea, fever, and cough in the 7 days preceding the survey [22]. Care seeking at a health facility was defined following DHS guidelines and was assessed only among parents of children who experienced illness.

Caregiver-level outcomes included intimate partner violence which was assessed among parents who were currently married, cohabitating, or in a relationship using items from DHS Domestic Violence Module [23] covering emotional, physical, and sexual abuse within the last 3 months. Among households with a mother-father structure, we also assessed whether caregivers reported equal involvement in decision-making about care for the young child including decisions related to feeding and medical care for illness [24]. Finally, caregivers’ mental health was assessed using the Hopkins Symptom Checklist-25 (HSCL-25), a measure of depression and anxiety (internalising) symptoms validated for use among adults in Rwanda [25] (α = 0.93). A mean score ≥ 1.75 was used to define likely clinical mental health problem.

Household-level outcomes related to hygiene practices were assessed using items from the DHS water, sanitation, and hygiene (WASH) module [22]. Indicators included access to clean water, safe treatment of water, and hand-washing facility with soap.

### Power calculation

Power calculations drew on previously conducted pilot studies [15] and estimated the required sample size for a 0.18 minimum detectable standardised effect size on parenting outcomes and child development outcomes for the 3- and 12-month follow-up period assuming power of 0.8 and a standard alpha level of *p <* 0.05. We used an estimated intraclass correlation of 0.03 for parent-child interactions based on pilot data. The ePW programme was being rolled out during the design phase of the programme, and we assumed based on estimates available to us it would be too scarce to constitute one half of an ideal sample size, so calculations were based on an assumption of 91 ePW clusters and 104 cPW clusters with five households per cluster to be assigned to treatment and control conditions. Because the target number of all ePW clusters did not exist, further adjustments were made, by adding combined clusters, and ultimately adding more cPW clusters to maintain power.

### Randomisation

**Fig. 2:**
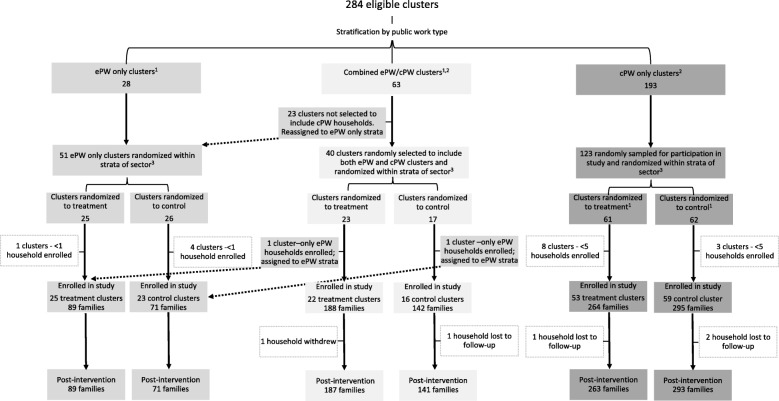
Cluster sampling strategy and flow chart of participants in the Sugira Muryango trial. cPW = expanded public works; cPW = classic public works. *Note:* Although each cluster had a 50% chance of being assigned to receive treatment, we were not guaranteed an equal number of treatment and control clusters because randomisation occurred within relatively small strata that sometimes contained an odd number of clusters

Families were enrolled between February and March 2018. Government staff in the three study districts provided lists of households participating in cPW or ePW. Families’ participation in VUP was determined by governmental policies and was not under the control of the researchers. Non-overlapping, geographically defined clusters were created comprising at least 30 families participating in the cPW programme or 10 families participating in the ePW programme, with some clusters containing both ≥30 cPW and ≥ 10 ePW households. Clusters were formed by the research team using detailed local maps by combining one or more contiguous villages such that one CBC could provide services to all participating families in the cluster. Villages within the same cluster were selected to be as close to each other and as far apart from other clusters as possible. Due to the relative scarcity of the ePW families, 100% of clusters containing at least 10 ePW families were sampled for participation in the study. Clusters which contained cPW families (including combined clusters containing ePW families) were randomly sampled for inclusion into our study until we reached our target sample size of ≥1040 households. Randomisation was performed by a data collection contractor and occurred at the cluster level within strata defined by public works type (ePW only, combined ePW/cPW, and cPW only) and geographic sector. Within strata, clusters were assigned random numbers and placed on a ranked list. The first half of clusters on the randomly ranked list were assigned to treatment. In case of an odd number of clusters per strata, a lottery was used to round the number assigned to treatment up or down. After assignment of the cluster, households were contacted by the data collection contractor and invited to participate in the study. Clusters were retained if at least five families in the cPW strata or at least one eligible family in the ePW strata enrolled. We retained 48 ePW-only clusters, 38 ePW/cPW clusters, and 112 cPW-only clusters (Cluster sampling strategy, Fig. 2). Neither the caregivers nor enumerators knew the family’s assignment status at the time of the baseline assessments. Enumerators were also not informed about the family’s assignment status during the post-intervention assessment although caregivers’ responses to fidelity questions about the programme following the assessment may have revealed their treatment status. In total, 1049 households were enrolled at baseline. After the randomisation *n* = 508 families were allocated to UC and *n* = 541 families were allocated to treatment. Baseline data collection occurred in May 2018 and post-intervention data were collected in August–September 2018.

### Statistical analysis

We compared trajectories of outcomes over time among families receiving the *Sugira Muryango* intervention with UC using linear mixed models for continuous outcomes and generalised linear mixed models with a logit link for binary outcomes. To account for clustering, we included random effects for randomisation cluster and child for outcomes assessed at the child level. For outcomes assessed at the caregiver level we included random effects for cluster and caregiver, and for outcomes assessed at the household level we included random effects for cluster and household. Following intention-to-treat analysis, we used chained equation imputations in STATA to account for missing data [26]. We report coefficients for the time-by-treatment interaction term and standardised effect sizes estimated based on the modelling results (Cohen’s *d* for continuous outcomes, odds ratios (OR) for dichotomous outcomes) with 95% confidence intervals. Analyses were conducted using StataSE version 15 (StataCorp, College Station, TX). Intraclass correlations can be found in Additional File 1, and further analyses examining whether a family’s enrolment in either ePW or cPW moderated intervention effects can be found in Additional File 2.

### Adverse events in intervention and control households

During the interval between baseline and post-intervention “risk of harm” cases were reported in 12 families (2.2%) in the intervention group and 12 families (2.4%) in the control group (see Additional File 3 for more information). These households were retained in the analyses under intention to treat.

### Analytic sample and demographics of the samples

Baseline data were collected on 1084 children and 1498 caregivers who were enrolled in the trial at baseline. Instances of loss-to-follow-up from baseline to the postintervention assessment was low (< 2.5%). More specifically, three households (0.3%), 36 caregivers (2.4%), and six children (0.5%) did not complete the post-intervention assessment and had post-intervention data imputed. Item-level missing data at both baseline and post-intervention were similarly low (< 1%). Descriptive statistics are provided in Table 3. Caregivers ranged in age from 18 to 79 years and were most frequently the biological mother (*n* = 950), the biological father (*n* = 433), or a grandparent (*n* = 96). Sixty-four percent (*n* = 953) of the caregivers were married or cohabitating. At enrolment, 61% of the families reported high levels of food insecurity and 48% of the children were stunted as defined by a standardised height-for-age (HAZ) score at or below -2SD of the reference population in accordance with WHO growth standards [27].

**Table 3:** Descriptive statistics of study participants at enrolment. Continuous variables reported as [mean (SD)] and binary variables reported as [frequency (%)]

	CLASSIC PUBLIC WORKS (cPW)		EXPANDED PUBLIC WORKS (ePW)	
	Sugira Muryango + cPW	cPW only	Sugira Muryango + ePW	ePW only
**HOUSEHOLDS (*N*** **= 1049)**	***n*** **= 374**	**n = 374**	***n*** **= 167**	***n*** **= 134**
**High food insecurity**	239 (63.9%)	229 (61.2%)	104 (62.3%)	70 (52.2%)
**CHILDREN (*N*** **= 1084)**	***n*** **= 386**	***n*** **= 384**	***n*** **= 173**	***n*** **= 141**
**Average age in months**	21.0 (8.14)	21.8 (8.6)	20.8 (8.2)	22.3 (8.4)
**Health status and wellbeing**				
Stunted (HAZ < 2)	184 (47.7%)	178 (46.4%)	85 (49.1%)	75 (53.2%)
Wasted (WHZ < 2)	13 (3.4%)	9 (2.3%)	8 (4.6%)	2 (1.4%)
Underweight (WAZ < 2)	63 (16.3%)	71 (18.5%)	30 (17.3%)	27 (19.1%)
Screens positive, disability or developmental delay	110 (28.6%)	111 (29.0%)	57 (32.9%)	38 (27.1%)
Any violent punishment	184 (47.7%)	180 (47.0%)	83 (48.0%)	59 (41.8%)
**CAREGIVERS (*N*** **= 1498)**	***n*** **= 555**	***n*** **= 564**	***n*** **= 211**	***n*** **= 168**
**Average age in years** ***[range]***	34.5 (9.7) [18–79]	35.7 (10.3) [19–75]	36.3 (10.6) [18–79]	37.5 (12.7) [18–84]
**Marital Status**				
Single, separated, divorced, widowed	171 (30.8%)	166 (29.43%)	117 (55.5%)	91 (54%)
Married/cohabitating	384 (69.2%)	398 (70.6%)	94 (44.5%)	77 (45.8%)
**Relationship with child**				
Biological mother	341 (61.4%)	338 (59.9%)	152 (72.0%)	119 (70.8%)
Biological father	179 (32.3%)	183 (32.4%)	44 (20.9%)	27 (16.1%)
Adoptive mother	2 (0.4%)	1 (0.2%)	0 (0.0%)	0 (0.0%)
Stepfather	1 (0.2%)	4 (0.7%)	0 (0.0%)	5 (3.0%)
Stepmother	1 (0.2%)	0 (0.0%)	0 (0.0%)	0 (0.0%)
Aunt/uncle	3 (0.5%)	2 (0.4%)	0 (0.0%)	0 (0.0%)
Grandparents	28 (5.0%)	36 (6.4%)	15 (7.1%)	17 (10.1%)
**Educational Attainment**				
No school/Don’t know	112 (20.2%)	132 (23.4%)	60 (28.4%)	38 (22.6%)
< 6 years	275 (49.5%)	252 (44.7%)	97 (46.0%)	88 (52.4%)
≥ 6 yrs. Primary	88 (15.9%)	89 (15.8%)	26 (12.3%)	21 (12.5%)
Secondary/vocational school	80 (14.4%)	91 (16.1%)	28 (13.3%)	21 (12.5%)
**Health and safety**				
Screens positive, disability	60 (10.8%)	63 (11.2%)	36 (17.1%)	26 (15.5%)
Screens positive, depression or anxiety	275 (49.5%)	248 (44.0%)	117 (55.5%)	83 (49.4%)
Maternal victimisation violence, last three months^a^	78 (39.8%)	73 (35.3%)	15 (29.4%)	15 (36.6%)
Paternal perpetration violence, last 3 months^a^	38 (21.2%)	41 (22.3%)	10 (23.3%)	4 (12.5%)

^a^ among mothers (*n* = 495) and fathers (*n* = 438) who are married or cohabitating

*HAZ* Height-for-age, *WHZ* weight-for-height, *WAZ* Weight-for-age

## Results

Results from the mixed models are shown in Table 4. Unadjusted baseline and post-intervention means for continuous outcomes and frequencies for binary outcomes can be found in Additional File 4.

**Table 4:** Model-based estimates and effect sizes for primary child development outcomes

Outcomes	Mixed model difference in difference estimates^**a**^	
	Estimated coefficient (95% CI)	Effect Size Cohen’s d (95% CI)^**b**^ or Binary: OR (95% CI)
**CHILD DEVELOPMENT (*N*** **= 1084)**		
**ECD stimulation in the home**		
HOME [0–43] (continuous)	3.85 (3.20, 4.50)	*d = 0*.87 (0.74, 0.99)
OMCI [0–57] (continuous)	3.06 (1.57, 4.56)	*d =* 0.29 (0.17, 0.41)
FCI (ECD activities) (continuous)	1.25 (1.01, 1.48)	*d = 0*.73 (0.60, 0.86)
**Child nutrition, health and safety**		
Dietary Diversity [0–7 food groups] (continuous)	0.45 (0.26, 0.64)	*d =* 0.35 (0.22, 0.47)
Diarrhoea prevalence (%)	−0.28 (−0.67, 0.11)	0.76 (0.51,1.11)
Diarrhoea care seeking (%) ^c^	1.49 (0.66, 2.31)	4.43 (1.95, 10.10)
Fever and cough prevalence (%)	− 0.18 (− 0.56, − 0.19)	0.83 (0.57, 1.21)
Fever and cough care seeking (%) ^d^	1.19 (0.60,1.77)	3.28 (1.82, 5.89)
**Child caretaking practices and child safety**		
Use of any harsh discipline (%)	−1.22 (−1.67, − 0.76)	0.30 (0.19, 0.47)
Exclusive nonviolent discipline (%)	0.92 (0.16, 1.68)	2.50 (1.17, 5.34)
**CAREGIVER OUTCOMES (*N*** **= 1498)**		
**Caregiver mental health**		
Screens for internalising problems (%) ^e^	− 0.54 (− 0.96, − 0.13)	0.58 (0.38, 0.88)
**Shared decision-making**^**f**^		
Action when child sick (%)	0.72 (0.27, 1.18)	2.06 (1.31, 3.26)
What child eats (%)	0.35 (−0.18, − 0.88)	1.43 (0.84, 2.43)
**Intimate partner violence**		
Perpetration, male caregivers (%) ^g^	− 0.11 (− 0.97, 0.75)	0.90 (0.38, 2.12)
Victimisation, female caregivers (%) ^h^	−0.72 (−1.43, − 0.01)	0.49 (0.24, 1.00)
**HOUSEHOLD OUTCOMES (*N*** **= 1049)**		
**Water, hygiene and sanitation**		
Place with soap to wash hands (%)	0.86 (0.42, 1.31)	2.37 (1.52, 3.69)
Water treatment (%)	1.22 (0.77, 1.67)	3.39 (2.16, 5.30)
Accessing clean water (%)	0.65 (0.01, 1.29)	1.91 (1.01, 3.62)

*HOME* Home Observation for Measurement of the Environment, *OMCI* The Observation of Mother-Child Interaction, *FCI* Family Care Indicators

^a^ Coefficients and effect sizes represent the “difference-in-difference” or “time-by-treatment” interaction between the two groups

^b^ Cohen’s *d* estimated from the regression coefficient for continuous outcomes using the pooled standard deviation of the outcome at baseline

^c^ Among those with prevalent diarrhoea (*N* = 376 at baseline and *N* = 394 at post-intervention)

^d^ Among those with prevalent fever or cough (*N* = 595 at baseline and *N* = 707 at post-intervention)

^e^ Scored ≥1.75 on the Hopkins Symptom Checklist-25 Questionnaire

^f^ Among married or cohabitating mothers and fathers (*N* = 913)

^g^ Among male caregivers reporting a current intimate partner at baseline (*N* = 450)

^h^ Among female caregivers reporting a current intimate partner at baseline (*N* = 523)

### Positive parenting and responsive care

Compared with UC, children receiving *Sugira Muryango* experienced improvements in caregiver engagement scored on the HOME, the OMCI, and the FCI. Improvements on the HOME inventory were 3.9 points greater among intervention families compared with UC (Coefficient = 3.85, 95% CI: 3.20, 4.50; Cohen’s *d* = 0.87, 95% CI: 0.74, 0.99). Improvements on the OMCI total score were 3.1 points greater among intervention families than UC (Coefficient = 3.06, 95% CI: 1.57, 4.56; Cohen’s *d* = 0.29, 95% CI: 0.17, 0.41). The increase in stimulating caregiving activities (FCI) was 1.2 activities greater in intervention families compared with UC (Coefficient = 1.25, 95% CI: 1.01, 1.48; Cohen’s *d* = 0.73, 95% CI 0.60, 0.86).

### Dietary diversity, child health, care seeking, and hygiene

Pre- to post-intervention, families receiving *Sugira Muryango* reported a significant increase of 0.45 food groups in children’s dietary diversity compared to UC (Coefficient = 0.45, 95% CI: 0.26; 0.64, Cohen’s *d* = 0.35, CI: 0.22, 0.47). Pre- to post-intervention the prevalence of acute childhood illnesses was unchanged; however, at post-intervention, the improvement in odds of seeking care for child diarrhoea were 4.4 times the higher in families receiving *Sugira Muryango* relative to UC (Coefficient = 1.49, 95% CI: 0.66, 2.31; OR = 4.43, 95% CI: 1.95, 10.10) and 3.3 times higher in seeking care for child fever in families receiving *Sugira Muryango* relative to UC (Coefficient = 1.19, 95% CI: 0.60, 1.77; OR = 3.28, 95% CI: 1.82, 5.89).

### Violence and harsh discipline

Following the 12-module intervention, the odds of exposure to harsh discipline decreased 70% more in families receiving *Sugira Muryango* compared to UC children (Coefficient = − 1.22, 95% CI: − 1.67, − 0.76; OR = 0.30, 95% CI: 0.19, 0.47). Moreover, the odds of being exclusively exposed to nonviolent forms of discipline increased 2.5 more for children in *Sugira Muryango* families compared to UC (Coefficient = 0.92, 95% CI: 0.16, 1.68; OR = 2.50, 95% CI:1.17, 5.34). *Sugira Muryango* was also associated with a 51% decrease in the odds of females reporting victimisation to intimate partner violence (Coefficient = − 0.72, 95% CI: − 1.43, − 0.01); OR = 0.49, 95% CI: 0.24, 1.00). We did not observe intervention related differences in the changes in father reports of intimate partner violence perpetration.

### Parental mental health and shared decision-making

The intervention was associated with significant improvement in anxiety and depression symptoms among caregivers receiving *Sugira Muryango* compared to UC (Coefficient = − 0.54, CI: − 0.96, − 0.13; OR = 0.58, 95% CI:0.38, 0.88). Dual-caregiver dyads receiving *Sugira Muryango* did not show increased shared decision-making regarding child feeding, but had twice the increase in odds of jointly deciding what to do when the child was sick (Coefficient = 0.72, 95% CI: 0.27, 1.18; OR = 2.06, 95% CI: 1.31, 3.26).

### Water, hygiene and sanitation

*Sugira Muryango* households also had 2.4 times greater improvement in odds of engaging in handwashing with soap (Coefficient = 0.86, 95% CI 0.42, 1.31; OR = 2.37, 95% CI: 1.52, 3.69) and 3.4 times greater improvement in odds of engaging in safe water treatment (Coefficient = 1.22, 95% CI: 0.77, 1.67; OR = 3.39, 95% CI: 2.16, 5.30) following the intervention compared with UC. Intervention households also had almost twice the odds in improvement in access to clean water in the intervention group relative to UC (Coefficient = 0.65, 95% CI: 0.01, 1.29; OR = 1.91, 95% CI: 1.01, 3.62).

## Discussion

*Sugira Muryango* was designed to address the needs of Rwanda’s most vulnerable families. Through home visiting, we involved a range of family members, including fathers, in nurturing care. We observed that active coaching, play, alternatives to harsh discipline and violence, and encouragement of family strengths can help vulnerable households create a better home and care environment for young children drawing on formal and nonformal resources. Results indicate that this brief (12–16 week) intervention led to improvements in caregiving practices related to child development including parent-child interactions and stimulation, nutrition, care seeking, and reduced violence. The observed effect sizes for changes in ECD-related parent behaviours fall within the range found in other home-visiting ECD interventions in LMICs. For example, our effect size for the HOME (*d =* 0.87) is comparable to effect sizes reported in previous studies in Pakistan [4] (*d =* 0.30), Uganda [19] (*d =* 1.1) and Bangladesh [5] (*d =* 0.55 to *d =* 0.68), and our effect size for the OMCI (*d =* 0.29) is comparable to that reported in Pakistan (*d =* 0.20 to 0.80) [4]. The observed effect size for dietary diversity (*d =* 0.35) is also comparable to effect sizes (*d =* 0.54) in a parenting intervention in Uganda [19] and in Bangladesh (*d =* 0.40) [5]. Although we did not see improvement in the prevalence of acute childhood illness in the intervention group, we did see improvements related to care seeking if children were sick. The absence of improvements in children’s health status was somewhat surprising given the impact of the intervention on caregiver behaviours that are known to improve child health including dietary diversity and improved hygiene. Two factors may explain the lack of improvement in child health. First, given the four-month interval between the baseline and post-intervention assessment, the season changed from rainy season (April/May) to dry season (August/September), which may impact the prevalence of childhood disease. A second explanation may be that children age over the course of the intervention and assessments and may become less prone to illness both in the intervention and the UC group as they age. *Sugira Muryango* was also associated with reductions in family violence—reduced use of harsh punishment practices and victimisation of mothers by intimate partner violence. Moreover, increased shared decision-making about what to do if a child was sick was indicative of increased father involvement in childcare.

Limitations must be noted. First, in this brief assessment period, we did not explore physical and cognitive development outcomes; these will be examined at 12-month follow up. Second, some measures relied on parent-report and could suffer from differential bias because parents who were exposed to the intervention may have been more aware of socially desirable responses. We also see a discrepancy between females’ reports of victimisation and males’ reports of perpetration of violence suggesting that male caregivers may not give accurate self-reports. Relatedly, we note that we limited this examination of intimate partner violence to victimisation of mothers and perpetration by fathers although we recognise other forms of intimate partner violence exist. On the other hand, a strength of the study is that key outcomes related to nurturing care, such as the OMCI and the HOME were reported by a blinded, trained observer. Third, *Sugira Muryango* was delivered to vulnerable households categorised as extremely poor, as defined by eligibility to a social protection programme offered to Ubudehe 1 households in Rwanda. The extreme level of poverty in these households may limit the generalisability of the results to other, less poor households. Future studies may examine how families of different socioeconomic status may benefit from the programme.

## Conclusion

Family home-visiting interventions like *Sugira Muryango* have an important role to play in promoting ECD and preventing family violence globally. The integration of ECD programmes and social protection agendas is a promising area for helping vulnerable children and families break intergenerational cycles of poverty and violence.

## Supplementary information

**Additional File 1.** Adverse Events

**Additional File 2.** Intra-class correlational

**Additional File 3 **Interaction by VUP status. Difference in the effects of the intervention emerged between cPW and ePW households on the HOME inventory (*p* = 0.002), shared decision making about what to do when a child is sick (*p* = 0.016), shared decision making about what a child eats (*p* = 0.020), and perpetration of violence (*p* = 0.031). A full table of results is in the supplemental material, Table S3. On the HOME inventory, we found that the intervention effect was greater in the ePW households compared with cPW households. For the shared decision-making questions, we found that intervention effects were limited to cPW households. For perpetration of violence we found that intervention effects were limited to ePW households.

**Additional File 4.** Baseline and post-intervention means. Unadjusted raw means from unimputed dataset.

## Data Availability

*Deidentified individual participant data will not be made available since this is an ongoing trial.* De-identified data will be available from the authors 6 months after collection of the 12-month follow-up. The dataset supporting the conclusions of this article are included within the article (and its additional files).

## Abbreviations

CBC: Community-based coach
DHS: Demographic and Health Surveys
cPW: Classic public works programme
ECD: Early child development
ePW: Expanded public works programme
FCI: Family Care Indicators
HOME: Home Observation for Measurement of the Environment
LMICs: Low- and middle-income countries
MICS: Multiple Indicator Cluster Survey
OMCI: Observation of Mother Child Interaction
UC: Usual Care
UNICEF: United Nations International Children’s Emergency Fund
VUP: Vision 2020 Umurenge Programme
WHO: World Health Organization
