# Supplementary material for: Promoting parent-child relationships and preventing violence via home-visiting: a pre-post cluster randomised trial among Rwandan families linked to social protection programmes
Source: BMC Public Health. 2020 May 6;20:621. doi: 10.1186/s12889-020-08693-7 (PMC7201751; doi:10.1186/s12889-020-08693-7)
Supplement: Supplementary file 1 — Additional File 1. Adverse Events [file 12889_2020_8693_MOESM1_ESM.docx]

***Intervention families***

In the intervention families five risk of harm cases—all related to caregiver suicidality—were identified during the baseline assessment. One risk of harm case—a violent attack by neighbours—occurred during the intervention. Six risk of harm cases occurred at midline in the intervention families. Of these, five were related to suicidality in the primary caregiver and one was related to severe intimate partner violence.

***Usual care families***

Four risk of harm cases were reported in the usual care families at baseline, of these three were related to caregiver suicidality and one was related to severe physical discipline towards children. Eight risk of harm cases were identified at the midline assessments in the usual care families, five related to caregiver suicidality, two related to severe intimate partner violence, and one was related to severe physical discipline towards the children.
