# Supplementary material for: Promoting parent-child relationships and preventing violence via home-visiting: a pre-post cluster randomised trial among Rwandan families linked to social protection programmes
Source: BMC Public Health. 2020 May 6;20:621. doi: 10.1186/s12889-020-08693-7 (PMC7201751; doi:10.1186/s12889-020-08693-7)
Supplement: Supplementary file 2 — Additional File 2. Intra-class correlational [file 12889_2020_8693_MOESM2_ESM.docx]

| **Outcomes** | **Intra-class correlation** |
| --- | --- |
| **CHILD DEVELOPMENT** |  |
| HOME (0-43) | 0.079 |
| OMCI (0-57) | 0.066 |
| FCI (0-6) | 0.046 |
| Dietary Diversity (0-7 food groups) | 0.050 |
| Diarrhoea prevalence (%) | 0.053 |
| Diarrhoea care seeking (%) | 0.112 |
| Fever and cough prevalence (%) | 0.032 |
| Fever and cough care seeking (%) | 0.080 |
| Use of any harsh discipline (%) | 0.027 |
| Exclusive nonviolent discipline (%) | 0.029 |
| **CAREGIVER OUTCOMES** |  |
| Screens for internalizing problems (%) | 0.042 |
| Action when child sick (%) | 0.037 |
| What child eats (%) | 0.020 |
| Perpetration, male caregivers (%)^3^ | 0.032 |
| Victimization, female caregivers (%) | 0.037 |
| **HOUSEHOLD OUTCOMES (N=1,049)** |  |
| Place with soap to wash hands (%) | 0.073 |
| Water treatment (%) | 0.077 |
| Clean water (%) | 0.341 |
