# Supplementary material for: Promoting parent-child relationships and preventing violence via home-visiting: a pre-post cluster randomised trial among Rwandan families linked to social protection programmes
Source: BMC Public Health. 2020 May 6;20:621. doi: 10.1186/s12889-020-08693-7 (PMC7201751; doi:10.1186/s12889-020-08693-7)
Supplement: Supplementary file 3 — Additional File 3 Interaction by VUP status. Difference in the effects of the intervention emerged between cPW and ePW households on the HOME inventory (p = 0.002), shared decision making about what to do when a child is sick (p = 0.016), shared decision making about what a child eats (p = 0.020), and perpetration of violence (p = 0.031). A full table of results is in the supplemental material, Table S3. On the HOME inventory, we found that the intervention effect was greater in the ePW households compared with cPW households. For the shared decision-making questions, we found that intervention effects were limited to cPW households. For perpetration of violence we found that intervention effects were limited to ePW households. [file 12889_2020_8693_MOESM3_ESM.docx]

| **Outcomes:**  Continuous reported as adjusted mean Binary reported as % | **Classic Public Works (cPW) Post-intervention** | | | **Expanded Public Works (ePW) Post-intervention** | | | **Test of differences^1^** |
| --- | --- | --- | --- | --- | --- | --- | --- |
|  | **Treatment** | **Control** | **Test for significant change among cPW households** | **Treatment** | **Control** | **Test for significant change among ePW households** |  |
| **CHILD DEVELOPMENT (N=1,084)** |  |  |  |  |  |  |  |
| **ECD stimulation in the home** |  |  |  |  |  |  |  |
| HOME [0-43] | 28·9 | 25·5 | <0·001 | 28·9 | 25·0 | <0·001 | 0·002 |
| OMCI [ 0-57] | 43·0 | 41·8 | 0·012 | 43·8 | 41·3 | <0·001 | 0·104 |
| FCI [0-6] | 4·62 | 3·49 | <0·001 | 4·55 | 3·15 | <0·001 | 0·579 |
| **Child nutrition, health and safety** |  |  |  |  |  |  |  |
| Dietary Diversity [0-7 food groups] | 3·50 | 3·00 | 0·001 | 3·36 | 3·09 | 0·001 | 0·296 |
| Diarrhoea prevalence (%) | 30% | 34% | 0·083 | 37% | 36% | 0·964 | 0·373 |
| Diarrhoea care seeking (%^)2^ | 79% | 56% | 0·013 | 90% | 56% | 0·001 | 0·294 |
| Fever and cough prevalence (%) | 66% | 69% | 0·174 | 71 | 66 | 0·727 | 0·306 |
| Fever and cough care seeking (%)^2^ | 75% | 45% | 0·003 | 69% | 42% | 0·004 | 0·396 |
| **Child caretaking practices and child safety** |  |  |  |  |  |  |  |
| Use of any violent discipline (%) | 32% | 46% | <0·001 | 0·26 | 0·44 | <0·001 | 0·227 |
| Exclusive non-violent discipline (%) | 14% | 8% | 0·056 | 13% | 7% | 0·061 | 0·636 |
| **CAREGIVER OUTCOMES (N=1,498)** |  |  |  |  |  |  |  |
| **Caregiver mental health** |  |  |  |  |  |  |  |
| Screens for internalising problems (%) | 17% | 22% | 0·002 | 35% | 25% | 0·982 | 0·126 |
| **Shared decision making^3^** |  |  |  |  |  |  |  |
| Action when child sick (%) | 38% | 22% | <0·001 | 32% | 25% | 0·313 | 0·016 |
| What child eats (%) | 19% | 11% | 0·035 | 11% | 13% | 0·104 | 0·020 |
| **Intimate partner violence** |  |  |  |  |  |  |  |
| Perpetration, male caregivers (%)^3^ | 8% | 7% | 0·516 | 2% | 7% | 0·039 | 0·031 |
| Victimisation, female caregivers (%)^3^ | 16% | 21% | 0·056 | 19% | 33% | 0·460 | 0·864 |
| **HOUSEHOLD OUTCOMES (N=1,049)** |  |  |  |  |  |  |  |
| **Water, hygiene and sanitation** |  |  |  |  |  |  |  |
| Place with soap to wash hands (%) | 89% | 81% | <0·001 | 85% | 82% | 0·010 | 0·141 |
| Water treatment (%) | 59% | 33% | <0·001 | 77% | 36% | 0·372 | 0·826 |
| Clean water (%) | 0·97 | 0·97 | 0·487 | 98% | 98% | 0·003 | 0·066 |

^1^Assesses the significance of the “time-by-treatment-by-public works program” interaction; ^2^Among sick children only; ^3^ Among married or cohabitating caregivers only
