# Supplementary material for: Promoting parent-child relationships and preventing violence via home-visiting: a pre-post cluster randomised trial among Rwandan families linked to social protection programmes
Source: BMC Public Health. 2020 May 6;20:621. doi: 10.1186/s12889-020-08693-7 (PMC7201751; doi:10.1186/s12889-020-08693-7)
Supplement: Supplementary file 4 — Additional File 4. Baseline and post-intervention means. Unadjusted raw means from unimputed dataset. [file 12889_2020_8693_MOESM4_ESM.docx]

|  | **Baseline** | | | | | | **Post-Intervention** | | | | | |  |
| --- | --- | --- | --- | --- | --- | --- | --- | --- | --- | --- | --- | --- | --- |
|  | **Control** | | | **Sugira Muryango** | | | **Control** | | | **Sugira Muryango** | | | |
| Continuous reported as mean and standard deviation Binary reported as frequency (%) | N | Mean  / % | SD | N | Mean  / % | SD | N | Mean  /% | SD | N | Mean/ % | SD | |
| **CHILD DEVELOPMENT** |  |  |  |  |  |  |  |  |  |  |  |  | |
| **ECD stimulation in the home** |  |  |  |  |  |  |  |  |  |  |  |  | |
| HOME (Continuous) | 525 | 24.85 | 4.82 | 559 | 24.57 | 5.00 | 522 | 25.34 | 4.54 | 556 | 28.91 | 4.27 | |
| OMCI (Continuous) | 525 | 40.78 | 10.77 | 559 | 39.31 | 10.61 | 522 | 41.63 | 11.07 | 556 | 43.22 | 10.09 | |
| FCI (ECD Activities) (Continuous) | 525 | 3.03 | 1.75 | 559 | 2.98 | 1.76 | 522 | 3.40 | 1.77 | 556 | 4.60 | 1.47 | |
| **Child nutrition, health and safety** |  |  |  |  |  |  |  |  |  |  |  |  | |
| Dietary Diversity [0-7 food groups] (continuous) | 525 | 2.78 | 1.33 | 559 | 2.76 | 1.31 | 522 | 3.02 | 1.29 | 556 | 3.46 | 1.26 | |
| Diarrhoea prevalence (%) | 525 | 32.95 |  | 559 | 36.31 |  | 522 | 37.55 |  | 556 | 35.61 |  | |
| Diarrhoea care seeking (%)^3^ | 173 | 55.49 |  | 203 | 55.67 |  | 196 | 52.04 |  | 198 | 75.76 |  | |
| Fever and cough prevalence (%) | 525 | 53.52 |  | 559 | 56.17 |  | 522 | 66.09 |  | 556 | 65.11 |  | |
| Fever and cough care seeking (%)^4^ | 281 | 65.12 |  | 314 | 65.92 |  | 345 | 45.50 |  | 362 | 67.13 |  | |
| **Child caretaking practices and child safety** |  |  |  |  |  |  |  |  |  |  |  |  | |
| Use of any harsh discipline (%) | 512 | 45.31 |  | 544 | 47.24 |  | 518 | 45.75 |  | 553 | 29.66 |  | |
| Exclusive nonviolent discipline (%) | 433 | 8.78 |  | 455 | 9.01 |  | 521 | 11.90 |  | 551 | 19.42 |  | |
| **CAREGIVER OUTCOMES** |  |  |  |  |  |  |  |  |  |  |  |  | |
| **Caregiver mental health** |  |  |  |  |  |  |  |  |  |  |  |  | |
| Screens for internalising problems (%) | 732 | 45.00 |  | 766 | 51.17 |  | 715 | 34.26 |  | 747 | 32.53 |  | |
| **Intimate partner violence** |  |  |  |  |  |  |  |  |  |  |  |  | |
| Perpetration, male caregivers (%)^7^ | 255 | 34.90 |  | 258 | 39.14 |  | 239 | 33.89 |  | 242 | 28.51 |  | |
| Victimisation, female caregivers (%)^8^ | 220 | 20.45 |  | 227 | 21.14 |  | 208 | 16.83 |  | 217 | 16.58 |  | |
| **Shared decision-making** |  |  |  |  |  |  |  |  |  |  |  |  | |
| Action when child sick (%) | 484 | 33.80 |  | 489 | 33.53 |  | 473 | 28.54 |  | 479 | 38.83 |  | |
| What child eats (%) | 484 | 18.59 |  | 489 | 20.00 |  | 473 | 18.81 |  | 479 | 24.22 |  | |
| **HOUSEHOLD OUTCOMES** |  |  |  |  |  |  |  |  |  |  |  |  | |
| **Water, hygiene and sanitation** |  |  |  |  |  |  |  |  |  |  |  |  | |
| Place with soap to wash hands (%) | 507 | 72.19 |  | 541 | 65.99 |  | 505 | 77.82 |  | 539 | 84.79 |  | |
| Water treatment (%) | 508 | 33.46 |  | 541 | 34.57 |  | 505 | 39.01 |  | 539 | 60.67 |  | |
| Accessing clean water (%) | 508 | 80.31 |  | 539 | 75.32 |  | 504 | 84.52 |  | 538 | 85.69 |  | |
